# Space, body, time and relationship experiences of recess physical activity: a qualitative case study among the least physical active schoolchildren

**DOI:** 10.1186/s12889-015-2687-0

**Published:** 2016-01-06

**Authors:** Charlotte Skau Pawlowski, Henriette Bondo Andersen, Tine Tjørnhøj-Thomsen, Jens Troelsen, Jasper Schipperijn

**Affiliations:** 1 Institute of Sports Science and Clinical Biomechanics, University of Southern Denmark, Campusvej 55, 5230 Odense M, Denmark; 2 Centre for Intervention Research in Health Promotion and Disease Prevention, National Institute of Public Health, University of Southern Denmark, Øster Farimagsgade 5a, 1353 Copenhagen K, Denmark; 3 The National Institute of Public Health, University of Southern Denmark, Øster Farimagsgade 5 A, 2, 1353 Copenhagen K, Denmark

**Keywords:** Physical activity, Children, School recess, The four existential lifeworlds, Participant observation, Participatory photo interviews

## Abstract

**Background:**

Increasing recess physical activity has been the aim of several interventions, as this setting can provide numerous physical activity opportunities. However, it is unclear if these interventions are equally effective for all children, or if they only appeal to children who are already physically active. This study was conducted to explore the least physically active children’s “lived experiences” within four existential lifeworlds linked to physical activity during recess: space, body, time, and relations.

**Methods:**

The study builds on ethnographic fieldwork in a public school in Denmark using a combination of participatory photo interviews and participant observation. Thirty-seven grade five children (11–12 years old) were grouped in quartiles based on their objectively measured daily physical activity levels. Eight children in the lowest activity quartile (six girls) were selected to participate in the study. To avoid stigmatising and to make generalisations more reliable we further recruited eight children from the two highest activity quartiles (four girls) to participate.

**Results:**

An analysis of the least physically active children’s “lived experiences” of space, body, time and relations revealed several key factors influencing their recess physical activity: perceived classroom safety, indoor cosiness, lack of attractive outdoor facilities, bodily dissatisfaction, bodily complaints, tiredness, feeling bored, and peer influence.

**Conclusion:**

We found that the four existential lifeworlds provided an in-depth understanding of the least physically active children’s “lived experiences” of recess physical activity. Our findings imply that specific intervention strategies might be needed to increase the least physically active children’s physical activity level. For example, rethinking the classroom as a space for physical activity, designing schoolyards with smaller secluded spaces and varied facilities, improving children’s self-esteem and body image, e.g., during physical education, and creating teacher organised play activities during recess.

## Background

Physical activity (PA) in childhood is associated with a multitude of short- and long-term health benefits by its preventive effects on numerous physical conditions, and ability to stimulate cognitive performance and mental wellbeing [1–5]. Despite the benefits of PA, a significant number of children in Denmark and other Western countries do not reach the recommended levels of PA per day [6], and PA typically decreases from being a child to being an adolescent [7, 8]. Since PA patterns in early life are likely to track into adulthood the importance of promoting PA among children is widely recognised [9–11].

School recess is a key setting to provide opportunities for children to be physically active because of its potential to reach and influence a large number of schoolchildren with different backgrounds [12–14]. Studies have also shown that recess can provide one of the largest contributions to children’s overall PA [15–17]. Recess can be a valuable contribution to the overall school-day physical activity, particularly for the least physically active children, as they are found to be more physically active during school hours than after school [14, 17, 18].

Increasing schoolyard PA during recess has been the aim of several interventions [19–24]. However, it is unclear if these interventions are equally effective for all children, or if they ‘just’ provide more opportunities for those who are already physically active. In general, there seems to be little knowledge about who the least physically active children are, although many studies describe girls and obese children as less physically active [14, 25–27]. The least physically active children cannot be considered as a homogenous group due to large individual differences [28]. Since we do not really know who the least physically active children are, we have little information on how they experience school recess and what influences their recess PA. Gaining in-depth knowledge of how the least physically active children experience recess can help develop tailored interventions beneficial to the least physically active children, and subsequently achieve improved health outcomes. Because the decline in PA is associated with the transition from childhood to adolescence we focused this study on children about to transition into adolescence (11–12 year old) to understand the mechanism of PA in this age group [7, 8].

### Theoretical framework

To gain insight into the least physically active children’s recess experiences a hermeneutic-phenomenological methodology was used as the underlying scientific basis for the study. Hermeneutic phenomenological research is the study of the lifeworld, that is, the meaning given to lived experiences. According to Husserl the lifeworld is an intuited and common world where we act without reflection [29]. Based on Husserl’s phenomenological thinking Van Manen has further developed a methodology of four existential lifeworlds to guide ones reflections on the phenomenon: lived space (spatiality), lived body (corporeality), lived time (temporality), and lived relations (relationality), which pervade the fundamental structure in the lifeworld of everybody independent of history and culture [30, 31].

In the current study, lived space refers to the feelings the children get in different spaces surrounding them in the school setting [30]. Lived body refers to how the children experience their body during recess and how it influences the way they feel and interact [30]. The children’s perception of time during recess is regarded as lived time [30]. Lived relations refers to how the children interact with each other during recess [30]. The four-dimensional perspective proves helpful as a guide for reflection in the research process [30] and facilitates an in-depth understanding of the explored phenomenon [32, 33], which in this study is the least physically active children’s lived experiences of recess PA.

### Aim

The aim of this study was to explore the least physically active children’s lived experiences of four existential lifeworlds linked to PA during recess: space, body, time, and relations.

## Method

### Setting

The study was carried out at a school in a rural lower middle class area in the western part of Denmark. At the school, 381 students, of which 99 % were ethnic Danes, were enrolled in junior (0–3 grade), middle (4–6 grade) or senior (7–9 grade) tiers. During the school day two breaks characterised by free play without any organised curriculum were offered: morning tea and a lunch break, lasting 30 min each. The junior students were required to stay outdoors during recess but the school had no outdoor recess policy for middle and senior tier students. The outdoor school grounds covered 13,311 m^2^ (35 m^2^ per child) and were divided into a paved schoolyard with play markings, a large grassy area with soccer fields, and a well-equipped playground for junior students.

The school was recruited from an existing schoolyard intervention study: The Activating Schoolyards Study aims to improve children’s opportunities to become physically active in the schoolyard during recess, particular for the least physically active schoolchildren [34]. The school is similar to many other Danish schools in terms of the type of school buildings, size, recess organisation, type of school ground and number of students [35].

### Recruitment and participants

For the current study, our primary interest was to study the least active 11–12 year old children from the two grade five classes at the school (5A and 5B). We used a sampling strategy based on objective measures of PA to classify the children’s PA level.

Prior to the current study, the baseline study of the Activating Schoolyards Study assessed children’s (grade 4–8) PA level objectively using accelerometers. All children were asked to wear accelerometers for seven consecutive days. During the measurement period the participants also completed an electronic survey, inquiring about background characteristics (e.g., height and weight) [34].

Across the two grade five classes 40 out of 47 children participated in the Activating Schoolyards Study, of whom 37 fulfilled the inclusion criteria of having at least one school day with 9 h of accelerometer data per day. In class 5A 19 children (13 girls) and in class 5B 18 children (11 girls) participated with 1–7 valid days of data. Based on their average daily minutes of moderate-to-vigorous physical activity (MVPA) the 37 children were divided into quartiles. Children within the lowest activity quartile were defined as the least physically active children and with a median of 40 min of MVPA per day they did not reach the recommended levels of 60 min of MVPA per day. Their median MVPA during recess was 5 min. Eight children (six girls) equally distributed between the two grade five classes were part of the lowest activity quartile. Among these, five were overweight based on the BMI threshold definition by Cole et al. [36], using self-reported height and weight measures (Table 1).

**Table 1:** Main characteristics of eight participants with 25 % lowest daily MVPA and eight children with 50–100 % highest daily MVPA, respectively

Characteristics	25 % lowest daily MVPA	50–100 % highest daily MVPA
	8 = X (100 %)	8 = X (100 %)
Minutes of daily MVPA		
<60 min	8 (100 %)	0 (0 %)
≥60 min	0 (0 %)	8 (100 %)
Minutes of recess MVPA		
<15 min	7 (87,5 %)	3 (37,5 %)
≥15 min	1 (12,5 %)	5 (62,5 %)
Class		
5A	4 (50 %)	4 (50 %)
5B	4 (50 %)	4 (50 %)
Gender		
Boys	2 (25 %)	4 (50 %)
Girls	6 (75 %)	4 (50 %)
BMI		
Overweight	5 (62,5 %)	2 (25 %)

*MVPA* moderate-to-vigorous physical activity

*BMI* body mass index

To avoid stigmatising and to make generalisations more reliable [37] we further recruited eight children (four girls) for participation in the data collection. The children in this group were equally divided between the two highest activity quartiles and they had a median of 87 min MVPA per day and a median of 16 min of recess MVPA. In total, 16 children participated in the study, eight from the lowest activity quartile and eight from the two highest activity quartiles (Table 1).

During the data collection the researcher in the field (the first author) did not know to which activity quartile the participants belonged in order to minimise the potential bias related to the researcher’s pre-understanding of their PA behaviour [30].

### Procedure

Data were collected during February and March 2015. The study employed an ethnographic approach [38] using a combination of participant photo interviews [39–41] and participant observation [42, 43]. These methods were chosen to follow the children closely and gain in-depth insight into the least physically active children’s lived experiences of PA during recess. Using multiple methods in researching children’s lived experiences during recess offered complimentary insights and understandings [44].

Participatory photo interviews (also called photo elicitation) is a method that uses photographs taken by the participants prior to the interview as a tool during the interview [40]. In our study, 16 children were interviewed individually. The interviews began with exploring their reflections on recess using the photos they had taken [41, 45]. We used the photos to stimulate dialogue, provide nuances, trigger memories [44–46], and reduce the authority of the researcher [47, 48] in line with the new paradigm of childhood [49].

Two days prior to the interview the children were sent a text message to their mobile phone saying: *“Please take three photos with your mobile phone of what you are doing during today’s recess”.* The children were instructed to send the photos to a teacher who forwarded all photos to the first author. The interviews took place at various times during the data collection period and lasted approximately 30 min per child. All interviews were recorded using an iPad mini®. An interview guide (Table 2) helped to focus on the four dimensions of the lifeworld during the interview [30] and to cross check data between children [50].

**Table 2:** The interview guide used for the participatory photo interviews

Initial questions linked to the photos
● How do you experience recess?
● What do the photos show?
● Why did you take these photos?
● Was it difficult to decide what kind of photos you wanted to take? Why/why not?
● What are you doing in the photo? Do you do that often?
● Who are you together with in the photo?/Are you doing it alone? Why?
● Where are you in the photo? Why are you there?
Supporting questions linked to the four lifeworlds
Lived space:
● Where do you like to go during recess? Why? Are you mostly there?
● Do you miss places to go during recess?
Lived body:
● Do you like to use your body during recess? Why/Why not?
● How do you like to use your body during recess? (Examples)
● How do you experience your own body?
Lived time:
● How do you experience time during recess?
● Do you like the short or the long recess periods most? Why?
● Do you keep an eye on the time during recess? Why/Why not?
Lived relations:
● What is a good class? Are you part of a good class? Why/Why not?
● How do you experience your class during recess?
● Are you playing with someone during recess? Why/Why not? Who?
● Are you together with children from outside your class during recess? Why/Why not? Who?

Participant observation is a method with roots in traditional ethnographic research and it is accomplished through varying degrees of observation and participation in the study community’s daily activities [42]. In current study, the first author was present at the school 1–2 days each week during the 2 months of data collection following the two classes (5A and 5B). Observing in two classes provided an opportunity to follow children in different class cultures. The observations took place both during free-play activities during recess and during teacher-controlled activities during lessons to get a more complete picture of their school day. To be able to pursue a phenomenon the observations were driven by an open approach to the explored field [51].

It was important to be aware that an adult researcher who attempts to understand children’s culture cannot pass unnoticed as a member of that group [43]. Acceptance into the world of children is highly challenging because of the obvious differences between adults and children in terms of cognitive and communicative maturity, power, and physical size [52]. This difference excluded a fully participating role in the children’s school life [53]. The researcher’s position was what Spradley calls “moderate participation” [42] where the researcher did not take initiatives directed at the children during observations such as starting a play, mediating in a conflict, or tying shoelaces. However, the researcher was careful of not being too passive. She followed the children around and hung out with them during recess which provided the chance to overhear intimate exchanges giving the researcher insights into the unknown [54].

### Ethics

The school principal approved the study and the parents from all 16 children invited to participate provided a written informed consent. If a child featured in photographic material the parents provided written, informed consent for further use of the photo as part of research material for dissemination and publication. All interviews were conducted as a confidential conversation between researcher and child. However, if a child had disclosed that he or she was at risk of harm, then the researcher had a duty to pass this information on to a professional (e.g., a teacher) who could protect the child [55].

According to the Danish National Committee on Health Research Ethics formal ethical approval was not required as the project was not a biomedical research project. Data management and data security procedures with regards to this study were approved by the Danish Data Protection Agency (2013-41-1900). The study adheres to the RATS guidelines for reporting qualitative studies.

### Analysis

After the data collection was completed the first author received information on who of the 16 participating children were the least physically active children. All field notes were reviewed and information on the children’s activity quartile was added. The 16 audio-recorded interviews were transcribed verbatim by the first author to aid recall and ensure accuracy [56]. The analysis was guided by the four lifeworld existentials: lived space, lived body, lived time, and lived relations. The four existentials were used as the analytical themes throughout the whole analysis. To ensure consistency, the first author manually coded each interview. Field notes and interview transcripts were analysed as a whole to explore the true nature of the phenomenon. The photos taken by the participants prior to the interview were used as supporting material during the analysis.

The analyses were primarily focused on the least physically active children. The first step focused on identifying meaning units for each of the least physically active children to be able to reconstruct a personal core story [30]. In the second more detailed analysis the meaning units of each least physically active child were tentatively grouped to capture the meaning of the experiences across different children. Finally, the grouped meaning units were compared to data from the other eight children to find similarities and differences between the least physically active and other children.

## Results

The lifeworld existentials of lived space, lived body, lived time and lived relations were used to identify the least physically active children’s lived experiences of PA during recess. In the following quotes, real names are replaced with aliases.

### Recess space

Most of the least physically active children experienced that the classroom was a pleasant place and they remained there during recess. These children expressed a strong affiliation to the classroom calling it *“our”* room and they explained that special norms and codes of behaviour, only understood by the children attending the class, were connected to the room making it a safe place to stay in. It was important to them that they could close the door and not be interrupted by children from other classes, as the following quote with a girl from class 5B attests:

Julie: *It’s a cosy place [the classroom], and it’s where you belong because you are here all day having all your lessons here.*

Interviewer: *Why is it important to be someplace during recess were you have a sense of belonging?*

Julie: *Because you know the place and you can do what you want to do in that place without being disturbed or others being irritated by you.*

The classroom was described as a quiet place for sedentary activities during recess and it was in here that most of the least physically active children were observed being immersed in a book, a drawing or a computer game. The classroom was also experienced as a social environment among many of the least physically active children. Togetherness and talking with classmates were two concepts strongly associated with the classroom:*”It’s the most silent place, you can say, and if you sit in there and read, then your friends are also often in there and you sit talking”* (Maria, class 5B)*.*

**Fig. 1:**
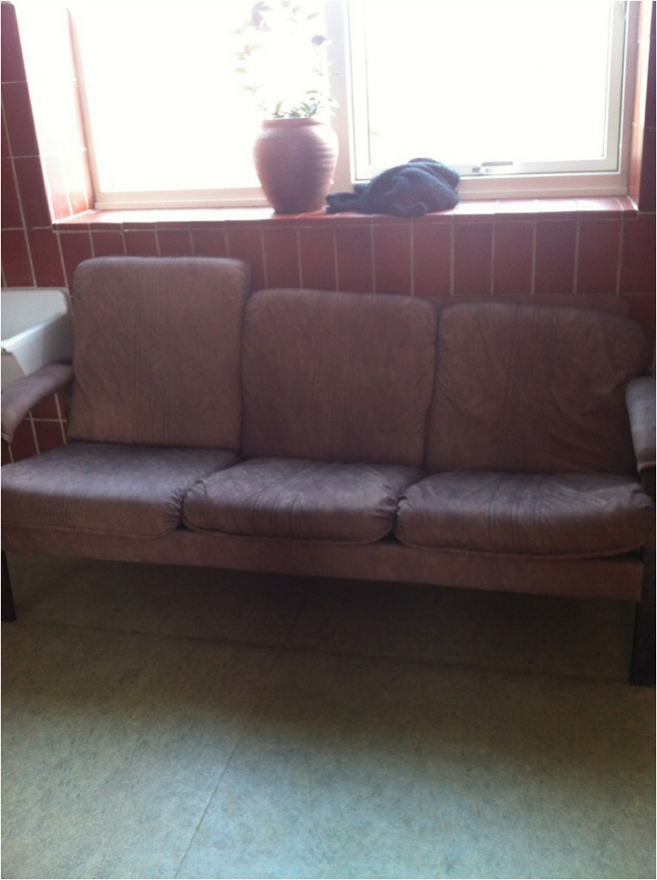
Anna’s photo of a couch in the corridor where she preferred to stay during recess talking with her friends

Some of the least physically active girls also favoured smaller secluded areas fitted with seats for talking and socialising such as a couch in the corridor or a seating arrangement in a corner of the library. The girls perceived these smaller areas as cosy and relaxing places where they could talk about girl-stuff or read a book undisturbed (see Fig. 1).

Some children told that they were mostly indoors because they perceived a lack of attractive outdoor facilities, as expressed by a girl from class 5A:

Olivia: *I miss things to do out in the schoolyard, some swings for instance*

Interviewer: *Do you prefer to stay outdoors or indoors?*

Olivia: *If there are things to do I prefer to stay outdoors*

Interviewer: *So, at the moment what do you prefer?*

Olivia: *I prefer to stay indoors*

Only one boy and one girl among the least physically active children preferred to stay outdoors during recess. They were at the field playing soccer like the majority of the most physically active children, and did not express an innate affiliation to the classroom.

### Recess body

**Fig. 2:**
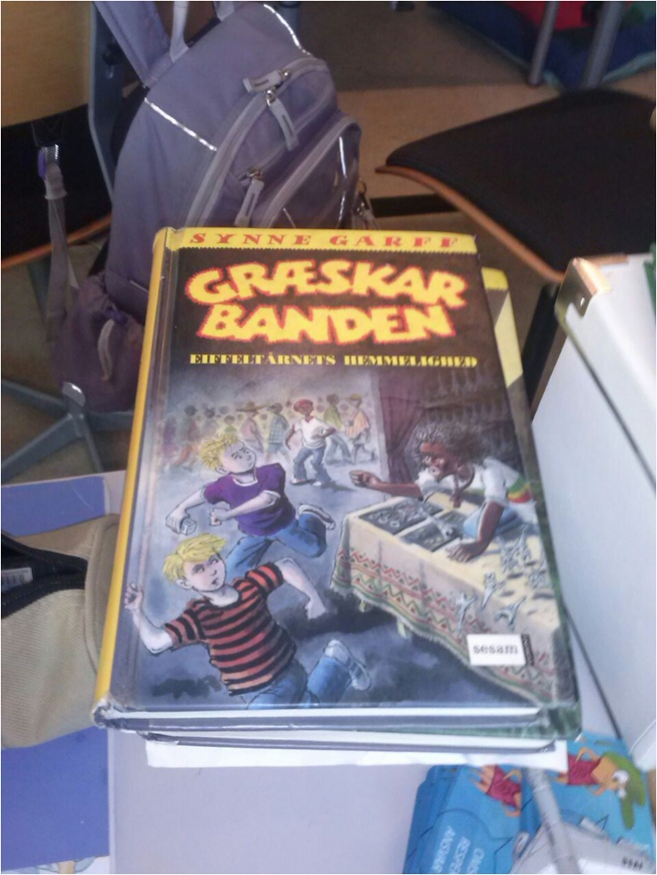
Albert’s photo of a stack of books on his desk

The least physically active children were aware of the benefits of having a *“fit”* and *“good-looking”* body. Particularly the least physically active overweight girls were aware that their body did not correspond to the common body ideal. They disliked their body and wanted to lose weight: *“I don’t think mine [body] is really beautiful, to be honest. At my confirmation party people should not look at me because I’m chubby, but because I look beautiful in my dress”* (Anna, class 5A). Feelings of body dislike seemed to make them choose recess activities not requiring bodily skills and performing, such as playing computer games, reading books, painting, listening to music and hanging out talking (see Fig. 2). In contrast, the most physically active children expressed that they mastered bodily skills such as *“being fast runners”, “good kickers”* or *“being flexible”*.

Some of the overweight children also expressed a feeling of being out-of-breath when using their body physically, or they explained that they were inactive because of *“injured knees”*, *“an injured toe”, “stomach pain”* or *“a headache”*: *“Often I have a headache and nausea or something like that. Then I can only sit or lay down”* (William, class 5A). The bodily complaints linked to overweight explained why some of the least physically active children mainly did sedentary activities during recess. However, not all overweight children complained about their body. We also observed an overweight boy being highly physically active playing soccer during recess.

Tiredness was another bodily explanation reported to inhibit the least physically active children in being physically active during recess. Repeatedly they mentioned episodes where they had been tired because of too little sleep, which hindered PA: *“I want to join but I get tired really quickly. I haven’t slept well. Sometimes I don’t sleep well”* (Albert, class 5B). In line with this some of the least physically active children also expressed feeling mentally tired after lessons and needing to clear their head during recess by *“relaxing”*. Their lived body experience differed from that of most of the physically active children who described a feeling of being *“hyper”;* an internal unrest in their body during lessons that had to be released by PA during recess, as expressed below:

*“Sometimes I can feel that I have been sitting still for a long time and then I really need to move my body. It’s like I can feel it in my legs, they start shaking because I have been sitting still too long and then I know when we get a break. I have to go for a walk in the schoolyard or play soccer and that’s very pleasant because then I know my legs will not start shaking in the next lesson”* (Jane, class 5A).

To reduce their restlessness some of the more active children were sitting on big balls during lessons, rocking back and forth.

### Recess time

Objectively, recess has a quantifiable time length. However, the children’s subjective perception of time differed. Generally, recess was verbalised as free time where they *“had fun”* or *“had a good time”* because they could do what they wanted to do, in contrast to the lessons, and for that reason recess was often experienced as a period where time went fast. In relation to this, many of the children’s experiences of lived time in the recess context were akin to feeling “timeless”. Feeling timeless implies the loss of objective time; an experience of being fully immersed in the moment. The least physically active children described playing computer games, talking and reading, as activities that could make them loose track of time during recess.

Some children wanted to utilise the “fast going” recess period optimally. Particularly the physically active children who played soccer used the time prior to recess to plan their activity to get the full potential out of the recess period, as described in a field note excerpt from a lunch situation in class 5B:

The teacher starts reading loudly from a book while the children sit quietly eating their lunch. Simon [a boy from the physically active group] suddenly exclaims:”Oh, you are so nice” addressed to the teacher. A boy asks:”why do you say that?” Simon responds:” She allowed us to have recess 1 min earlier”. A couple of minutes before the bell rings, commotion starts in the classroom. The teacher stops reading and say: “calm down, I have promised Simon that you can have your break a little earlier today. It has something to do with getting the soccer goals”. She continues reading. Simon stands up from his chair and walks out in the corridor. Some of the other boys turn around and crane their neck to get a better view of the soccer field from the window. Simon enters the classroom again loudly stating: “they are already gone” [some children from class 5A are off to the soccer field]. Simon goes back to his desk and sits down on the edge of his chair constantly looking at a clock hanging at the wall. The teacher closes the book and immediately Simon and many of the other boys run out of the classroom.

Planning recess activities so intensely was not observed among the least physically active children.

We observed that a few of the least physically active children were sitting passively while observing other children’s play. These children expressed that recess sometimes felt long. Explanations for this perceived elongated recess were *“feeling bored”* or *“having a bad day”*, which anchored them in objective time by constantly looking at a clock. They clearly expressed that their perception of time was connected with togetherness: *“When I feel bored time is going so slow but together with my friends time goes fast”* (Albert, class 5B).

### Recess relations

The children believed that having friends contributed to the enjoyment of activities. When children were asked why they participated in activities the most common responses were *“because my friends do”* or *“I like being together with my friends”.*

Both classes were divided into groups of indoor-staying and outdoor-staying children. Most children in the least physically active group stayed indoors socialising with each other during recess: *“We are a bit split up during recess because most are out playing soccer or whatever they are doing, actually I don’t know where they are, but we are just sitting indoors talking”* (Maria, class 5B). Primarily girls were socialising indoors. The division in outdoor-staying and indoor-staying children seemed strongly peer influenced, but without inducing gender segregation. One of the least physically active boys felt he belonged to the indoor-staying group and he took part in the girls’ conversations. Oppositely, one of the least physically active girls belonged to the outside-staying group mostly consisting of boys playing soccer. Her explanation for staying outside playing soccer was that she had just got a new soccer-playing girlfriend who encouraged her to play soccer during recess. In the least physically active group, the boy who played soccer also had encouraged a friend to start playing soccer, as highlighted below:

*I can’t get bored with playing soccer. It’s also me who has persuaded one of my friends to start playing soccer. I said: “Come outside and try to play during recess then you can see how enjoyable it is”. Then he started to go out and play and then he actually found it awesome and now he has started in a soccer club and soccer is one of his main interests* (William, class 5A).

**Fig. 3:**
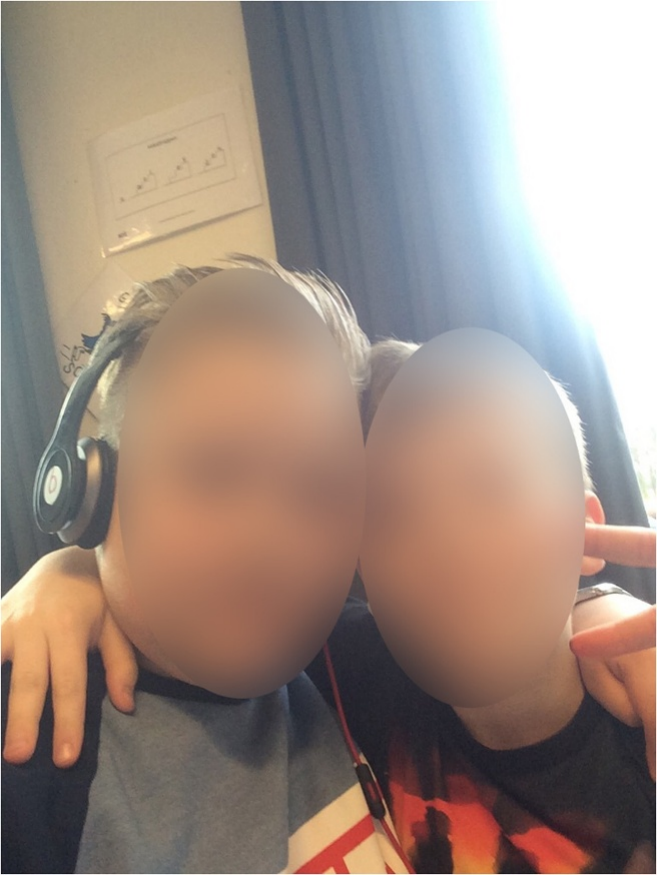
William’s photo of him and his best friend living next to each other. They were together during recess despite having different interests. The parents of the children gave consent to publish the picture

Many of the children expressed that they felt they belonged to a group of children coming from the same geographical area because it was easier to be friends with children living nearby. Many of these lived relations were friendships that had started prior to their school attendance and these existing friendships seemed to influence their recess behaviour (see Fig. 3).

In class 5A we observed one of the most physically active girls doing sedentary activities during recess with her friends from the same geographical area (girls from the low activity quartile) even though she had other interests, as outlined in the following quote:

Jane: *We have a pleasant time braiding each other’s hair, sitting talking or eating. It’s just cosy [during recess].*

Interviewer: *Who are “we”?*

Jane: *Me, Emma, Laura and Olivia. They’re my best friends.*

Interviewer: *Why are they your best friends?*

Jane: *We have been friends since our first years. Olivia and I are “baby friends”, that is what we call it, because we attended the same day care.*

Interviewer: *Do you also have the same interests?*

Jane: *No, we have a little different interests*

In general, the least physically active children seemed to have good relations to their classmates even though many of them were part of smaller groups keeping their distance from each other. In contrast, many of the most physically active children were part of bigger groups across the two grade five classes playing soccer on the field, which often triggered conflicts about winning. The indoor-staying boy explicitly explained that he stayed indoors during recess because he wanted to avoid the conflicts between the soccer-playing boys.

## Discussion

The present study set out to contribute to the current literature on children’s recess PA by examining the least physically active children’s lived experiences of PA during recess. Using the four existential lifeworlds, lived space, lived body, lived time, and lived relations facilitated a detailed and in-depth understanding of the explored phenomenon.

Six of the eight least physically active children stood out from the rest in regards to lived space during recess preferring indoor space, in particular their classroom. Indoor activities during recess are linked with sedentary activities [14, 57], so staying indoors might influence their recess PA. In regards to lived space “classroom safety”, “indoors cosiness” and “lack of attractive outdoor facilities” were perceived as key factors for remaining indoors during recess. According to Van Manen, children need to feel comfortable or intimate in the space [30]. It seemed as if the classroom was the secure inner sanctuary where these children felt protected, similar to being at “home”. A place where they could be themselves without being confronted with how good or bad they were at performing certain things [58]. In contrast, the wide-open outdoor space seemed to make them feel exposed, whereas the outdoor playing children possibly felt free in this setting. In line with this, Rasmussen found that not all children related to official places provided by adults, such as playgrounds during recess, but that they also related to informal places, unnoticed by adults [59]. In our study, most of the least physically active children related to their classroom seat, a couch or a seating arrangement in the library. Another study found that almost half of the 175 children included in the study, mostly girls, wanted the option of staying indoors [60]. In contrast, a study by Darmody et al. found that most children identified the schoolyard as their “favourite” place associated with fun and relaxing [61]. However, that study did not investigate experiences among different subgroups of children.

In regards to lived body, “bodily dissatisfaction” (both regarding bodily aesthetics and skills), “bodily complaints” and “tiredness” were perceived as key factors related to preferring sedentary activities during recess. Other studies have also found body-related barriers to PA among adolescents, such as dissatisfaction with body image and lack of competences [62, 63]. In our study body-related concerns were associated with sedentary behaviour in particular for the overweight girls. One of the overweight boys also preferred sedentary indoor activities, but this was explained by the play on the soccer field being too competitive. The masculine ideal tends to imprint to boys from a young age what it means to be a man. Boys are told that being muscular and competitive are ambitious qualities [64]. For boys not conforming to these ideals, a lack of self-esteem can be a consequence [65, 66]. One boy from the group of least physically active children, who was also overweight, did prefer to play soccer during recess. However, he had difficulties being physically active because of fatigue and somatic pain, as did the overweight girls. In a review by Stankov et al., fatigue and physical discomfort were also found as barriers for being physically active among overweight adolescents [62]. We also found that children complained about bad sleep habits demotivating them from doing recess PA. In line with our study, other studies on children have found associations between inadequate sleep and sedentary time [67] as well as physical inactivity [68].

The least physically active children’s temporal perspective showed that recess time was perceived as speeded up when they were enjoying themselves and slowed down when they were feeling bored. In line with our study, a study by Mulryan-Kyne showed that the majority of children typically experienced recess as a fast going enjoyable time [69]. In another study, children mentioned being able to enjoy games and physical exercise as positive features of recess [70], similar to most of the high activity children in our study. In contrast, we found that the least physically active children were more focused on sedentary socialising activities as positive features of recess. However, sometimes some of the least physically active children were observed being sedentary because they felt bored and alone, which made recess time feel elongated, as explained by Van Manen [30]. Consequently, feeling bored was perceived as a temporal factor related to being sedentary among some of the least physically active children.

Socialising with friends was perceived as important during recess. This is in line with Blatchford et al. who found that recess first and foremost was a social event [71]. A study found that, for many children, school was the only setting in which opportunities for learning to negotiate and manage conflicts as well as form new friendships with a wide range of peers from their own and other classes existed [69]. However, in our study most of the least physically active children were close friends and peer relations had been established in kindergarten. This seemed to impact the individual’s choice of recess activities. Typically, they preferred to sit in smaller groups socialising verbally, whereas most of the high physically active children were socialising while using their body. However, one of the least physically active girls was influenced by her peers to play soccer and one of the physically active girls was influenced by her peers to be sedentary during recess. Peer influence seemed to be both a facilitator and barrier to recess PA. Another study only found that peer influence was positively associated with PA [72]. However, that study did not explore different subgroups of children indicating that the negative association between peer influence and PA might relate to the low PA children.

### Implications for practice and research

Previous studies have reported that, on average, girls and obese children are less physically active [14, 25–27]. On the basis of current study we pose that treating the least physically active children as one homogeneous group is unproductive. Researchers and professionals working with children’s PA during school hours should be aware of the broad range of meanings and experiences linked to the four existential lifeworlds which are complex and interrelated. To increase PA among the least physically children a multi-component intervention is probably necessary.

Rethinking the classroom as a space for PA might be effective for some of the indoor staying children. For example, showing music videos on a screen was found to facilitate dancing in a previous study [28]. Designing schoolyards with smaller secluded spaces and varied PA promoting facilities seems recommendable to motivate children to go outside. This is supported by other studies claiming that more varied spaces [35] and activities in the schoolyard for those who do not want to play soccer will increase the overall PA level [16, 20, 23]. Also children’s attitude towards their own body seems to be an important factor in stimulating children to be physically active. Psychologists have suggested that the most important factors affecting body image are self-esteem and body control [73]. Several studies have shown that PA contributes to a significant increase in self-esteem in both boys and girls [74–76]. It is suggested that schools can play a vital role in improving children’s self-esteem and body image [64]. For example by focusing on the psychological mechanisms linked to using your body during physical education. Teacher organised play activities with less focus on competition and skills could be implemented to motivate more children to participate in schoolyard play. This is supported by research findings from a number of studies indicating that structured non-competitive activities with close supervision can promote cooperative play [77–79]. By participating in these activities, children also have the possibility to socialise and form new friendships.

### Strength and limitations

The systematic use of the four existential lifeworlds facilitated an in-depth understanding of the least physically active children’s lived experiences of recess PA. However, since we used the four existentials as an underlying framework both during data collection and data analysis it was important to be aware of the risk of getting a narrow-minded impression and to be conscious about the fact that the four existentials are a simplification that might not capture the whole lifeworld. Furthermore, the existentials were closely interconnected and the intention was not to separate the children’s experiences [80]. Therefore, we continually stepped back from the individual lifeworld-themes to take a more holistic look at the material.

The use of multiple methods strengthened the current study as it enriched the data and improved credibility of results [44]. Particularly, the use of a child participatory approach using photographs taken by the children was valuable to capture the phenomenon of children’s lived experiences of recess PA since self-directed photos can capture ordinary interactions of children’s daily lives, with the aim of uncovering meaningful content areas that, from an adult viewpoint, might be overlooked [48, 81].

Another strength of our study was the objective participant selection strategy that meant the researcher did not know who the least physically active children were throughout the data collection reducing bias based on pre-understandings, which gave her the analytical opportunity to be challenged in her views [30, 82]. Some of the children surprised us as we, based on the interview and observations, assessed them to be more physically active than they actually were. However, the objective sampling approach also gave us some challenges. Only 37 out of 47 children (78 %) in the two classes had 1–7 valid days of accelerometer data and could potentially be included in the study. More days of valid data would have been advisable for measuring the children’s PA level but would have reduced the amount of participating children further. Another limitation was that the children’s BMI was estimated from self reported height and weight. Moreover, the objective data were collected in June 2014 and in the current study data were collected in February and March 2015 and changes in the children’s lived experiences of recess PA might have occurred during this time period. However, the external environment remained identical (e.g., the children remained in the middle tier building, and class constellation and recess facilities were equal) and in both data collection periods the weather was variable with changeable temperatures.

## Conclusion

We found that Van Manen’s four existential lifeworlds provided a useful framework for acquiring an in-depth understanding of the essential aspects of the least physically active children’s lived experiences of recess PA. Within all four lifeworlds diverse experiences emerged. Due to the complexity of factors that emerged in this study we pose that treating the least physically active children as one homogeneous group is unproductive in future interventions. Based on this study, rethinking the classroom as a space for PA might be effective for some of the indoor staying children. Furthermore, designing schoolyards with smaller secluded spaces and varied PA promoting facilities is recommendable. Also, improving the least physically active children’s self-esteem and body image (e.g., during physical education) may positively influence the children’s motivation to be physically active. Finally, teacher organised play activities less focusing on competition and skills could be implemented to motivate more children to participate in schoolyard play.
